# Supplementary material for: Know your epidemic, know your response: Early perceptions of COVID-19 and self-reported social distancing in the United States
Source: PLoS One. 2020 Sep 4;15(9):e0238341. doi: 10.1371/journal.pone.0238341 (PMC7473541; doi:10.1371/journal.pone.0238341)
Supplement: S1 Table — (PDF) [file pone.0238341.s001.pdf]

S1 Table: Demographics characteristics of the sample

	Mean	Std
Male	0.483	0.500
Age	48.065	17.161
Bachelor degree or more	0.333	0.471
White	0.766	0.423
Black	0.128	0.334
Married	0.537	0.499
CA/NY/WA	0.222	0.416

Notes: The table shows the weighted mean and standard deviation of basic demographic characteristics. We use sample weights to make the survey representative of the U.S. population aged 18 and older. Data come from "Understanding America Study" (UAS) collected between March 10 and March 16, 2020.
