# Supplementary material for: Know your epidemic, know your response: Early perceptions of COVID-19 and self-reported social distancing in the United States
Source: PLoS One. 2020 Sep 4;15(9):e0238341. doi: 10.1371/journal.pone.0238341 (PMC7473541; doi:10.1371/journal.pone.0238341)
Supplement: S2 Table — (PDF) [file pone.0238341.s002.pdf]

S2 Table: Compare characteristics of respondents and non-respondents

	Respondents	Non-respondents	χ^2	p-value
Male	0.49	0.471	1.368	.242
Age	49.289	46.643	23.803	0.000
Bachelor degree or more	.349	.32	3.967	0.046
White	.771	.768	0.058	.81
Black	.121	.138	2.222	.136
Married	.567	.513	11.647	0.001
CA/NY/WA	.199	.081	160.665	0.000

Notes: The table shows the weighted mean of basic demographic characteristics for UAS participants who answered the survey by March 16 and for UAS participants who did not. We use sample weights to make the survey representative of the U.S. population aged 18 and older. The weights are different from those in the rest of the paper as they are constructed to make the whole UAS sample nationally representative. Data come from "Understanding America Study" (UAS) collected between March 10 and March 16, 2020.
