# Supplementary material for: Know your epidemic, know your response: Early perceptions of COVID-19 and self-reported social distancing in the United States
Source: PLoS One. 2020 Sep 4;15(9):e0238341. doi: 10.1371/journal.pone.0238341 (PMC7473541; doi:10.1371/journal.pone.0238341)
Supplement: S3 Table — (PDF) [file pone.0238341.s003.pdf]

S3 Table: Heterogeneity by Source of Information

	(1) Get Covid	(2) Die from Covid	(3) Excess Mortality	(4) Lose Job	(5) Out of Money	(6) Distancing
used Fox News	-0.037***	-0.009	-0.012***	-0.003	0.008	-0.023
	(0.009)	(0.009)	(0.004)	(0.010)	(0.014)	(0.019)
used CNN	0.041***	0.014**	0.014***	0.005	0.015*	0.120***
	(0.010)	(0.007)	(0.003)	(0.008)	(0.008)	(0.018)
Observations	5223	5222	5221	3214	5255	5247

Notes: The table reports the coefficients of linear regressions to show heterogeneity by source of information in 1) the chances of getting the virus within three months, 2) the chances of dying from the virus if infected, 3) excess mortality, 4) the chances of losing job within three months, 5) the chances of running out of money because of the virus within three months and 6) whether individuals refrain from at least one social activity. Used Fox News and CNN are 0/1 answers to the question "Which of the following information sources have you used to learn about the coronavirus in the past 7 days?". Control variables include age, gender, four education categories, state and day fixed effects. We use sample weights to make the survey representative of the U.S. population aged 18 and older. Robust standard errors are reported in parentheses. * $p < 0.1$, ** $p < 0.05$, *** $p < 0.01$. Data on perceptions and social distancing come from "Understanding America Study" (UAS) collected between March 10 and March 16, 2020.
