# Supplementary material for: Know your epidemic, know your response: Early perceptions of COVID-19 and self-reported social distancing in the United States
Source: PLoS One. 2020 Sep 4;15(9):e0238341. doi: 10.1371/journal.pone.0238341 (PMC7473541; doi:10.1371/journal.pone.0238341)
Supplement: S4 Table — (PDF) [file pone.0238341.s004.pdf]

S4 Table: Effects of Covid cumulative deaths on Covid perceptions and social distancing

	(1) Get Covid	(2) Die from Covid	(3) Excess Mortality	(4) Lose Job	(5) Out of Money	(6) Distancing
			Regression with state fixed effects			
US Deaths	0.002***	-0.000	0.000	0.002***	0.002***	0.006***
	[0.001,0.002]	[-0.001,0.000]	[-0.000,0.000]	[0.001,0.003]	[0.001,0.003]	[0.005,0.007]
			Regression with state and day fixed effects			
State Deaths	0.003	-0.002	-0.000	-0.002	-0.003	-0.005
	[-0.010,0.015]	[-0.008,0.004]	[-0.003,0.002]	[-0.010,0.006]	[-0.011,0.005]	[-0.015,0.005]
Observations	5272	5270	5269	3245	5301	5287

Notes: The table shows the effects of the log of the number of US and state of residence Covid-19 deaths on 1) the chances of getting the virus within three months, 2) the chances of dying from the virus if infected, 3) excess mortality, 4) the chances of losing job within three months, 5) the chances of running out of money because of the virus within three months and 6) whether individuals refrain from at least one social activity. The top panel shows regressions that include state fixed effects while the bottom panel shows regressions that include both state and day of interview fixed effects. State deaths are transformed using the inverse hyperbolic sine $log(x+\sqrt{1+x^2})$ which is very similar to log but allows for zeros. Additional controls include age, gender and four categories of education. We use sample weights to make the survey representative of the U.S. population aged 18 and older. 95% confidence intervals in squared brackets are calculated using standard errors clustered at the state level. * $p < 0.1$, ** $p < 0.05$, *** $p < 0.01$. Data on perceptions and social distancing come from "Understanding America Study" (UAS) collected between March 10 and March 16, 2020. Data on the number of Covid-19 deaths come from the Johns Hopkins University Center for Systems Science and Engineering.
