# Supplementary material for: Know your epidemic, know your response: Early perceptions of COVID-19 and self-reported social distancing in the United States
Source: PLoS One. 2020 Sep 4;15(9):e0238341. doi: 10.1371/journal.pone.0238341 (PMC7473541; doi:10.1371/journal.pone.0238341)
Supplement: Attachment — Submitted filename: Response to reviewers.pdf [file pone.0238341.s005.pdf]

Response letter to the reviewers' comments on manuscript submission PONE-D-20-12921 Entitled "Know Your Epidemic, Know Your Response: Early Perceptions of COVID-19 in the United States"

Dear Editor,
dear Reviewers:

Thank you very much for the valuable feedback on our manuscript PONE-D-20-12921 and giving us the opportunity to revise and resubmit our manuscript to PLOS ONE. The provided comments raised some good points and were very useful in helping us to improve the manuscript's quality.

This letter outlines our changes in the manuscript in response to the reviewers' comments and suggestions and provides specific answers to all issues raised in their reviews. For convenience, we first reproduce the reviewers' comments and then provide corresponding answers after each comment in italics.

Reviewer #1:

Overall assessment: The study seems to be very interesting. Appreciate the efforts taken by the researchers for a timely and pertinent COVID related article.

Authors' answer: Thank you very much for your positive feedback and appreciative comments.

Comment: The title of the study is catchy however it is not reflecting the variables properly thereby loosing the essence of the paper.

Authors' answer: As you correctly point out, the current title of our manuscript only mentions perceptions but our study also contains results on social distancing. We have therefore changed the current title of our manuscript to reflect that point. The revised title of our manuscript is now: "Know Your Epidemic, Know Your Response: Early Perceptions of COVID-19 and Self-Reported Social Distancing in the United States".

Comment: The study does mention about ethical committee permission and participant consent.

*Authors' answer: Thanks for pointing it out. We have added in the "Data and Method" section the following sentence that provides readers with information about ethical committee permission and participant consent: "*The online survey was approved by USC's Institutional Review Board, as part of the UAS and informed consent was obtained from all participants."

Comment: There is less clarity been given in the paper regarding variables under study.

Authors' answer: Thank you for this comment. We have improved the clarity of our presentation throughout the paper, and of the data/method component in particular. Our analysis contains six outcome variables: 1) chances of getting Covid-19, 2) chances of dying from Covid-19 conditioning on being infected, 3) excess mortality which is the product of 1) and 2), 4) chances of losing one's job because of Covid-19, 5) chances of running out of money within the next three months because of Covid-19 and finally 6) social distancing.

The following paragraph, which is taken from the paragraph in the middle of page 4 describes how variables 1, 2, 4 and 5 are defined:
"The risk perceptions variables used in the analysis are derived from the following questions: 1. "On a scale of 0 to 100 percent, what is the chance that you will get the coronavirus in the next three months?"; 2. "If you do get the coronavirus, what is the percent chance you will die from it?"; 3. "What is the percent chance that you will lose your job because of the coronavirus within the next three months?"; 4. "What is the percent chance you will run out of money because of the coronavirus in the next three months?" Respondents answered these questions using a scale from 0 to 100. In the analysis, we rescaled the answers by dividing by 100 to interpret the variables as probabilities. Question 3 was asked only to respondents who had a job at the time of the interview (N=3252)."

It is true that we do not specify in that paragraph how we defined variable 3 – "Excess mortality". We have therefore added a sentence in that regard, hoping that this clarifies your concern. The sentence we included in the paragraph is the following: "Using responses to questions 1) and 2), we derive a measure of excess mortality due to Covid-19 by multiplying the chances of getting infected with the chances of dying from the virus conditioning of being infected."

Finally, outcome variable 6 – social distancing—is defined in the paragraph that follows the one reported just above, which we reproduce here:
"We define a dichotomous measure of social distancing by exploiting the answers from the question "Which of the following have you done in the last seven days to keep yourself safe from coronavirus in addition to what you normally do?". We consider that a respondent was refraining from at least one social activity if the person admitted to have done at least one of the following: "Canceled or postponed travel for work", "Canceled or postponed travel for pleasure", "Canceled or postponed work or school activities", "Canceled or postponed personal or social activities", "canceled a doctor's appointment", "avoided contact with people who could be high-risk", "avoided public spaces, gatherings, or crowds", "avoided eating at restaurants" or "worked or studied at home".

Comment: The questionnaire its items were not fully comprehensive to reach a conclusive evidence.

Authors' answer: Thanks for your comment. Note that, related to your point above, we have added a sentence on how we derive the "excess mortality" measure so that all six outcome variables we use in our analysis are clearly defined in the text. Moreover, we have added more details in the "Data and method" section by reporting the Cronbach's Alpha of the different items that we use to construct our measure of social distancing. Cronbach's Alpha was equal to 0.83, which indicates good reliability of the items to measure the same construct –social distancing—(Nunnally, 1994).

Moreover, we also added a new section in the paper (Limitations section) in which we acknowledge the limitations of the social distancing measure but also justify our choice. We hope this answers your comment.

Comment: Validity and reliability of the questionnaire is not mentioned in the paper.

Authors' answer: Thank you for this comment. We added in the text two references that discuss in more detail the validity and reliability of the UAS questionnaire on Covid-19 we use in this study :

Bruine de Bruin and Bennet, Relationships Between Initial COVID-19 Risk Perceptions and Protective Health Behaviors: A National Survey

Bruine de Bruin, Age differences in COVID-19 risk perceptions and mental health: Evidence from a national US survey conducted in March 2020

In particular, from the second reference: "To obtain a nationally representative sample, UAS members were recruited from randomly selected U.S. addresses (Understanding America Study Recruitment Protocol, 2019), sampling probabilities were adjusted for underrepresented populations, and internet-connected tablets were provided to interested individuals if needed (Alattar, Messel, & Rogofsky, 2018). Address-recruited online panels tend to be better than opt-in online panels at achieving national representativeness (Tourangeau, Conrad, & Couper, 2013) and delivering high-quality data (Kennedy et al., 2020). Following the survey literature (Valliant, Dever, & Kreuter, 2013), poststratification weights were used to further align the present sample to the U.S. adult population regarding age, gender, race/ethnicity, education, and location (see https://uasdata.usc.edu/page/Weights)."

Additionally, we report the Cronbach's Alpha as a measure of reliability as pointed out above.

Comment: There is occasional typos and grammatical errors.

Authors' answer: Good point. All of us carefully went over the manuscript once more to correct the remaining typos and grammatical errors. We believe that this current version is free from any mistakes.

Comment: The figures were ambiguous.

Authors' answer: Sorry about that. It seems that there was an issue with the format of the file we appended to our manuscript. We have changed the file format of the figure, hoping that Figure 1 is now clearer and less blurry.

Comment: While assessing the perceptions it would be better if there is adequate number of items to investigate a behavioral response ;risk perception variable were assessed by using only a 4 item scale which lacks comprehensiveness of the information.

Authors' answer: It is true that risk perception in our analysis is assessed by four different items, so that our assessment of perceived risk due to Covid-19 might not be comprehensive enough. That being said, we are interested in this study in very specific risk perceptions, such as the risk of getting infected with Covid-19 or the risk of losing one's job because of the pandemic. It is therefore not clear to us why we would need several items that specifically ask about a specific risk perception such as the risk of getting infected, other than perhaps decreasing measurement error (as we all know, measurement error in dependent variables does not affect the estimates of our coefficients as long as that error is not correlated with control variables included in our model, which is plausibly the case in our specification). Moreover, single-item measures tend to have good predictive validity, as compared to multi-item measures (Diamantopoulous 2012 + Huang et al., 2020 see full reference in paper).

We nonetheless acknowledge the issue you raised in your comment and hence included a corresponding sentence in the limitation section.

Comment: Sample size should be mentioned in the top of each table.

Authors' answer: Thank you for this comment. We added the sample size on top of tables 1 and 2.

Reviewer #2:

Comment: This is an interesting paper and the topic is worthy of further discussion. My doubts are how the authors have called the data representative in the manuscript. They have given a comparison in the methodology as well which can be improved from a reader's perspective.

Authors' answer: Thank you for this comment. As mentioned in the text, the representativeness of the UAS study has been analyzed and extensively discussed in the literature, as evidenced by the references we included to support our claim. The new version of our manuscript now includes the reference to another study that provides further information about the representativeness of the UAS study:

"Bruine de Bruin, Age differences in COVID-19 risk perceptions and mental health: Evidence from a national US survey conducted in March 2020"

Kapteyn, A., Angrisani, M., Bennett, D., Bruine de Bruin, W., Darling, J., Gutsche, T., Liu, Y., Meijer, E., Perez-Arce, F., Schaner, S., Thomas, K., & Weerman, B. [The CESR COVID-19 Task Force] (2020). Tracking the Effect of the COVID-19 Pandemic on the Lives of American Households. Survey Research Methods, 14, 179-186.

We also would like to stress that the UAS data has been found to match the quality of the Health and Retirement Study (HRS), which is traditionally considered as a gold standard in social research (Angrisani, M., Finley, B.,& Kapteyn, A. 2019; Venti 2011). We included a statement in that regard in the manuscript.

Secondly the period of data collection was during the early stages of pandemic spread in the country and the same should be discussed while discussing the findings of the paper. The knowledge about pandemic and related behavior is bound to be affected by a number of confounders: baseline socio-economic parameters, financial difficulties during pandemic and the effect of social isolation. All these are dynamic and should be stated in the discussion section to enhance the context of study findings.

Authors' answer: We agree that what we describe refer to the early stages of the pandemic. We tried to emphasize this aspect from the title ("Early") and the abstract ("March 2020"). We also agree that things have probably changed a lot after this first period. To make the point more clear, we added a sentence in the Limitations section to stress that we should be cautious to extrapolate these findings to later stages of the pandemic.

Reviewer #3:

Overall assessment: This is a very interesting and well meaning piece of work, conducted in very scientific manner.

Authors' answer: Thank you for the positive assessment of our paper.

Comment: Abstract- You have not described the study methodology in the abstract; Kindly consider briefly describing the same in the abstract, especially when PLOS has flexibility with its word limit

Authors' answer: Thanks for your comment. We are now more specific about the sample we use in the paper and we also specify that we use fixed effects regressions for the analysis of COVID-19 cases. The rest of the analysis is just descriptive statistics which does not require any specific details on the methodology used.

Comment 2. Its hard to distinguish between the degree of social distancing which was due to behaviour change and self imposed and that due to stringent local governmental regulations. If the same can be segregated, I feel the same would strengthen the article, else that can go as study limitation.

Authors' answer: This is a very valid comment, thank you. In the Limitations section we added to our manuscript, we now acknowledge that the social distancing measure we use in our analysis is just not the result of self-imposed behavioral responses, but also the result of government regulations that might limit mobility and impose social distancing measures.. In the same paragraph, we also explain that the stay-at-home orders that were implemented to contain the spread of Covid-19 were all passed after our study period. Indeed, our analysis covers the period from March 10 to March 16, and governments at that time were mostly providing information to the population about the importance of hand-washing and social distancing for those with symptoms.

Comment: 3. In the similar lines, I suggested that you may consider correlation of the data on social distancing with stringency of local regulations on movement and other regulatory measures.

Authors' answer: Thanks for the suggestion. We are planning to explore these aspects in a future paper.

Comment: 4. I feel that you essentially need to improve the figure 1, which is quite blurred and uninterpretable

Authors' answer: Thank you for this comment. One of the reviewers raised a similar comment. As explained above, there was an issue with the file format.

Reviewer #4:

Comment: 1. Authors should be carefully classify the social distancing. If they have considered participant is following social distancing if participant answered “yes” for any one of the option, it may lead to overestimation. Many work related travels would have cancelled due to lockdown and restrictions on travel around the world. Participants would have cancelled work related travel due restrictive measures taken by governing body, but may not be following any of social distancing measures. This may lead to bias in the study.

Authors’ answer: We agree that this is an important concern. We now discuss the limitations of our social distancing measure in the limitation section we added to our manuscript. To partially alleviate these concerns, we explain that, at the early stages of the pandemic, strong mobility restrictions were not yet in place (at least in the United States). For this reason, our measure of social distancing shows a high degree of variation, with many US residents not reporting any behavioral response at that time (early March). We agree that the same measure of social distancing today would not be as informative.

Comment: 2. Without reading the methodology and results, it is difficult to understand Table 1. Complete the categories name in Table 1.

Authors’ answer: Right, we forgot! We now added the description of the variables in table 1.

Comment: 3. Figure 1 is not clear and could not make out the legends, which make it difficult to comment.

Authors’ answer: Thank you for this comment. One of the reviewers raised a similar comment. As explained above, there was an issue with the file format.

Comment 4. The authors need to adhere to STROBE statement for reporting observational studies.

Authors’ answer: In view of the various changes that we made to our manuscript following the useful comments of the reviewers (such as inclusion of a limitation section and more description of the study sample used), we are now in full compliance with the STROBE statement according to the checklist found here: https://www.strobe-statement.org/fileadmin/Strobe/uploads/checklists/STROBE_checklist_v4_combined.pdf

We have therefore added a corresponding sentence in the manuscript that says that the reporting of our study conforms to the STROBE statement (Von Elm et al., 2007).

Comment: 5. Introduction: At the end of introduction section, it is important to explicitly mention the objectives of the study.

Authors’ answer: The new version of our manuscript now includes at the end of the introduction section a few sentences that explain the objective of the study. The sentences we added are the following:

"In particular, the objectives of this study are to show the average levels of risk perceptions of getting infected with Covid-19, the mortality and economic risks associated with the pandemic as well as the variation in these risk perceptions by basic demographic characteristics. Similarly, the study presents average levels and heterogeneity in self-reported social distancing measures. Finally, our study explores how changes in individual risk perceptions and behavioral response correlate with changes in COVID-19 confirmed cases over time at the country and state level."

Comment: 6. Materials and Methods: Probability based sampling and providing tablet and internet to the respondents who do not have them adds crucial value to the study and reduces selection bias.
The Understanding America Study has nearly 8500 participants. The authors have used data from 5414. Explanation should be given as to how they were chosen. If 5414 were the number of respondents for this survey, then characteristics of non-respondents should be compared to respondents.

Authors' answer: Thanks for giving us the chance to clarify. To be precise, on March 10, 8815 UAS respondents were invited to take the survey. By the end of the field period (March 31), 7145 respondents had answered the survey for a response rate of 81%. Our study covers the period from March 10 to March 16. In that first week 5414 respondents had taken the survey. The additional 1731 respondents completed the survey between March 17 and March 31. We wanted to focus on the early stages of the pandemic before all the stay-at-home orders were issued.

The new version of our manuscript now includes a table in the Appendix (TABLE S2) that compares the characteristics of the UAS respondents who participated in the online survey during March 10th and March 16th with the characteristics of those who are enrolled in the UAS study but did not complete the online survey by March 16th.

As one could probably expect, there are statistically significant differences between the two groups, although they are not large in magnitude. The only exception in which the difference is somewhat large is in the difference in response rates between participants who live in states that were hit early during the pandemic versus others. Importantly however, we want to stress that the weights used in the analysis are calculated in such a way to make our study sample of 5414 respondents representative of U.S. residents.

Comment 7. Results:i. The first line in the results reads as follows: "U.S. residents perceived on average a 20% chance of getting infected with the coronavirus during the next three months". The table shows that there is considerable difference between mean (20%) and median (10%). It would be better to give SD and IQR to understand the distribution, to better interpret the results.

Authors' answer: Good point. We added the interquartile range to have a measure of the dispersion in risk perceptions.

Comment ii. The authors have discussed some of the results in this section of the manuscript. These needs to be included in the discussion section.

Authors' answer: Indeed we had an entire paragraph in the results section that was discussing the results. We now moved the whole paragraph in the discussion section. Thank you for this comment.

8. Discussion:

i. The discussion section needs to be in detail and include a summary the key results, explanation for the results that have been obtained.

Authors' answer: The discussion section now includes a summary of the key results.

ii. The number of cases and deaths in the one-week period during which the study was conducted rose rapidly. The authors need to elaborate how this could have affected perception of risk of infection and death.

Authors' answer: We agree that the time dimension is important. This is why we look at how perceptions change with the increase in COVID-19 cases and deaths. We find that the infection probability rose with the number of cases as well as the economic worries and social distancing. The probability of dying conditional on being infected is less dependent on the number of cases as it has to do more with the intrinsic lethality of the virus.

We also now stress in the limitations section that our findings refer to that particular week and cannot be applied to later periods.

iii. The authors explain the excess perception of mortality risk to pessimism among the people… Could this be because of the situation in Italy at that time.... A question examining how much of the perception of the individuals is related to the situation experienced by other countries must be included...

Authors' answer: We added a sentence in the discussion to reflect this point: "This pessimism was perhaps influenced by the high level of mortality experienced in other parts of the world such as in Northern Italy."

iv. The authors have not included strengths and limitations of the study

Authors' answer: We now included an entire section that explains the limitations of the study on page 13.

v. The authors have failed to explain how perception of the people would sway the response to COVID-19.

Authors' answer: We agree that our paper only analyzes the individual response to COVID-19 while it does not study how individual perceptions influence the policy response to COVID-19. This is a very relevant research question that should be explored in the future.

If your comment is about how individual perceptions shape individual's behavioral responses to COVID-19, our introduction section touches upon that and provides references to interested readers. A formal analysis of this topic is however beyond the scope of our current study.

Additional references

Bruine de Bruin and Bennet, Relationships Between Initial COVID-19 Risk Perceptions and Protective Health Behaviors: A National Survey

Bruine de Bruin, Age differences in COVID-19 risk perceptions and mental health: Evidence from a national US survey conducted in March 2020

Diamantopoulous, Journal of the Academy of Marketing Science et al 2012

Angrisani, M., Finley, B.,&Kapteyn, A. (2019). Can internet match high-quality traditional surveys? Comparing the Health and Retirement Study and its online version. In K. P. Huynh, D. T. Jacho-Chávez, & G. Tripathi (Eds.), The econometrics of complex survey data: Theory and applications (pp. 3–33). Emerald Publishing.

Kapteyn, A., Angrisani, M., Bennett, D., Bruine de Bruin, W., Darling, J., Gutsche, T., Liu, Y., Meijer, E., Perez-Arce, F., Schaner, S., Thomas, K., & Weerman, B. [The CESR COVID-19 Task Force] (2020). Tracking the Effect of the COVID-19 Pandemic on the Lives of American Households. *Survey Research Methods, 14,* 179-186.

Venti S. Economic measurement in the health and retirement study. In: Forum for Health Economics & Policy; vol. 14. De Gruyter; 2011
